# The communicative Umwelt for creative design, addressing the psychology of sustainability, to solve future global challenges

**DOI:** 10.3389/fpsyg.2026.1746896

**Published:** 2026-03-09

**Authors:** Claus-Christian Carbon

**Affiliations:** 1 Department of General Psychology and Methodology, University of Bamberg, Bamberg, Bavaria, Germany; 2 Research Group EPÆG (Ergonomics, Psychological Aesthetics, Gestalt), Bamberg, Bavaria, Germany; 3 Bamberg Graduate School of Affective and Cognitive Sciences (BaGrACS), Bamberg, Bavaria, Germany

**Keywords:** adaptation, creation, design, ecology, environment, innovation leadership, psychology, radical innovation

## Abstract

Design does not emerge solely from individual creativity but from ongoing interactions between humans and their Umwelt—the subjective, meaning-structured world through which environments are perceived, interpreted, and acted upon. This article develops the concept of a Communicative Umwelt as a psychologically grounded framework for sustainable design, specifying it as a system of entities, signals, channels, and feedback mechanisms that shape creative processes, user acceptance, and longer-term market dynamics. By operationalizing Umwelt beyond metaphor, the paper connects perceptual and cognitive psychology with design practice and sustainability-oriented innovation. The framework is situated in relation to adjacent literatures, including ecological psychology, design semiotics, participatory and systemic design, and sustainability transitions, and is distinguished by its focus on psychological meaning-making and feedback-driven transformation. Rather than advancing universal market claims, the article proposes mechanism-oriented pathways and boundary conditions under which locally embedded, context-sensitive design practices may foster sustainable consumption patterns. Sustainability is thus reframed not as a technical constraint but as an emergent outcome of communicative interactions between humans, artifacts, and socio-ecological systems, offering a theoretically informed basis for future empirical and comparative research.

## 1 The need for creative solutions

The call for creative solutions is a constant companion of designing new products. In the light of existential ecological challenges humankind faces today, this call for creative solutions has become even more intense. The excessive usage of primary material, energy and water and the growing quantities of worn out products and trash pose major environmental problems that cannot be solved by isolated particular interventions, but require creative holistic solutions based on a systematic global perspective.

## 2 The need for a psychological view

The natural resources of the Earth are by definition finite, and given today’s level of human activity, these resources are exploited in a clearly unsustainable way—at present, we consume natural resources 1.8 times (retrieved 14 February 2026) as fast as the Earth can renew them (Global Footprint Network, 2026).^1^ In addition, the excessive human-made pollution of nature could turn our planet into an inhabitable place (Vogel et al., 2019). In 2018, an apocalyptically sounding article was published and reached a wide audience as top news: “Domino-effect of climate events could move Earth into a ‘hothouse’ state” (Watts, 2018). The article referred to a recent scientific theory of so-called “Tipping Cascades” that could activate domino-like cascades of climate-relevant factors. These cascades are supposed to irreversibly increase the average temperature to a level that will close the door to a stabilized Earth pathway (Steffen et al., 2018). The authors, mainly having a background in climate and environment research, explicitly suggest that “a deep transformation based on a fundamental reorientation of human values, equity, behavior, institutions, economies, and technologies is required”—with one word: our situation is existential, and we need efforts that go much beyond what we have already thought and done.

In such an existential situation, with the world potentially standing on the edge of the abyss, we need support from the scientific discipline that typically takes care of such fundamental problems and offers solutions for emerging from crises: Psychology. Psychology, which will be my line of argumentation, can define social spaces and conditions that assist the creative process, provide techniques that help extract the best creative solutions, and offer methods for evaluating gained solutions, including their future acceptance and usage (Carbon, 2019).

I will illustrate the power of the psychological view by reference to the extreme example of contemporary ecological problems, but the general approach is applicable to smaller and less existential design needs as well. All in all, it is about creating the most adequate environment to address future challenges with the best and most adequate design. Therefore, we also have to understand the user of a product better in order to realize precisely what users’ needs and requirements are and how we can measure and even predict the acceptance of designed products of that innovative kind.

All my subsequent arguments will be based on the very general and very simple yet central insight that consumers, as well as designers, are humans—humans living in a psychological cosmos of general as well as specific needs, with typical ways of perceiving and understanding the world. This simple and seemingly self-evident insight must be reflected and translated into a systematic plan incorporating this critical psychological view by solid theory, methods, and data (Carbon, 2019).

## 3 Creativity

To assist in designing creative products, we must consider at least two types of interested parties. First, the user, and second, the designer. Both groups are embedded in a context of Zeitgeist and culture that trigger and shape specific mindsets. This means that both groups live and think in narrowed worlds, and, in fact, the boundaries of these worlds can hamper the designing of highly creative (and innovative) products. The solutions generated within the said restricted frame may meet fast adoption and good acceptance. However, they will not be substantially new and thus will not support a comprehensive rethinking and redefinition of common practices. However, taking a new perspective would be crucial for addressing existential problems. So how to empower people to leave this mental cage? There are several psychological techniques to address this challenge. The most important rule we must follow is: Prevent any “Denkverbote” (restrictions on free thought)! This means that, initially, we have to allow any kind of design ideas to be followed at least to a certain stage (see Gray et al., 2019), even IF colleagues and experts have good arguments as to why these ideas will not work and even IF we are not talking about our special expertise field (see Garfield, 1990). This inhibition of immediate rejection is mandatory to be able to follow unpaved pathways and also to build upon these ideas (and potential failures) to generate substantially new designs. The encouragement of creating ideas without the need to prove their feasibility is explicitly requested by many creativity techniques but is often neglected or even prevented in practical contexts (Isaksen and Gaulin, 2005), especially when groups are strictly hierarchically organized (Benbasat and Lim, 1993). One prominent example is the technique of brainstorming: Brainstorming is a seemingly simple and frequently practiced technique commonly construed as “thinking about the thing a bit more freely”. But that is not the whole story, in fact. Brainstorming in the specific sense of a group creativity technique as initially developed and proposed by Osborn (1963) explicitly calls for deferring judgment and pushing quantity. That way, social inhibition shall be prevented as it would end up in a restricted number of ideas, with most of them not being genuinely creative. While brainstorming is a classical group technique, there are a number of similar techniques that can be applied by single persons to unlock creative barriers, for instance “Art streaming,” “Remembrance,” or “Rightbraining.” Such techniques often start with dreaming or loosely thinking about a problem. With another technique called “Talk streaming” people are invited to talk about anything or anyone without the need to be consistent or logically correct. Altogether, these creativity techniques aim to get rid of the common thinking schemes, they ask for abstracting from usual solutions and they try to find new ways of perceiving a problem. Operating with subconscious processing helps very much in this regard, especially when we get into the favorable state of experiencing “flow” (Csikszentmihalyi, 1991) where many ideas can be created in a concise amount of time and with much more joy (O'Keefe and Linnenbrink-Garcia, 2014).

**Figure 1:**
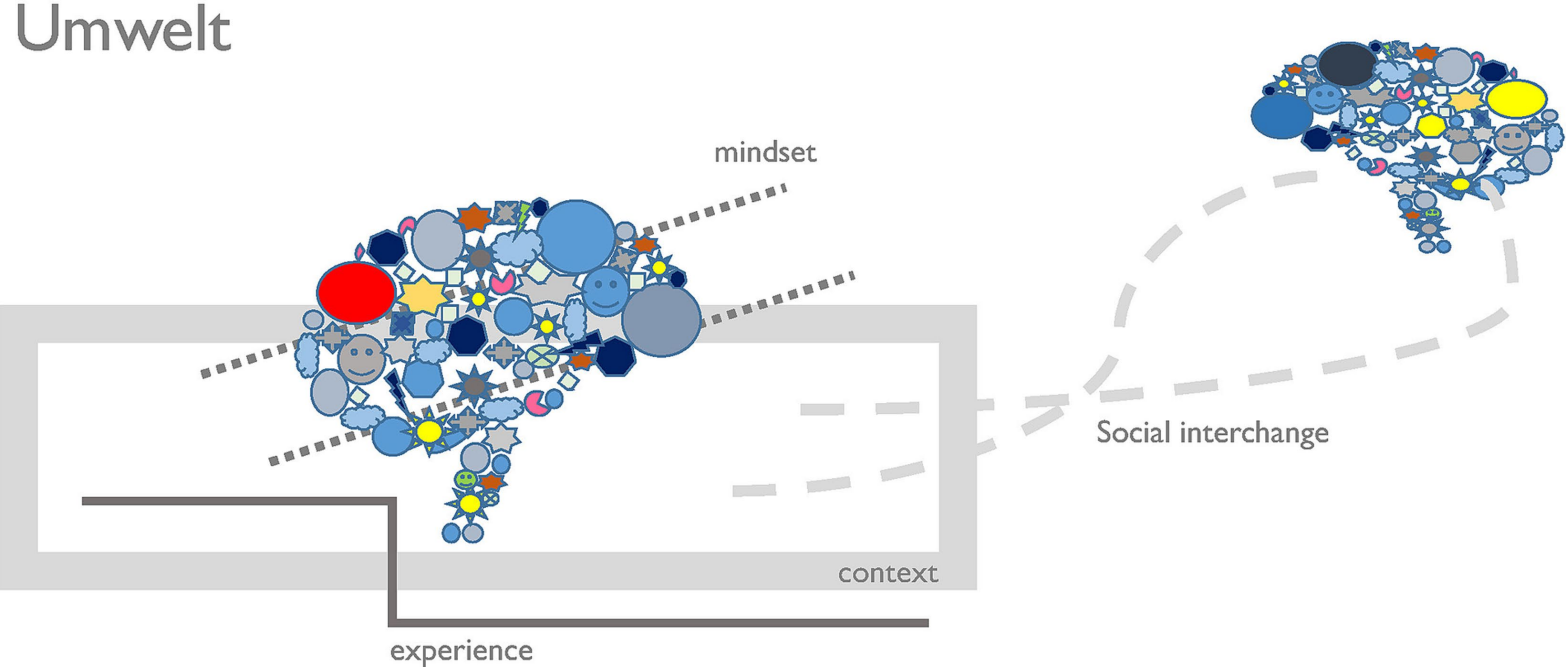
The (communicative) *Umwelt* which has to be created before we are in the right mental setting to generate substantially new creative ideas that help us to develop creative products for very challenging requirements.

In order to develop ideas that are not only creative or different from usual solutions, but which do also head towards sustainable products that fit into a larger system context of ecological and energy-saving approaches, we need more than the common techniques. Additionally, we need to activate an Umwelt (von Uexküll, 1909) that is triggering favorable mindsets for creating productive ideas. At a further stage, but not evidently initially, we need experience and expertise with regard to the impact of involved material and the interplay with the ecosystem. Figure 1 provides a schematic depiction of the Umwelt where also the social interchange with others is shown in order to activate group solutions and to be able to employ creative techniques that demand the involvement of others. For instance, when using the “Checklist technique,” the counterpart repeatedly asks “Why” something is needed, why it works, why we use this, and ultimately, why we need it at all. Asking these why-questions, similarly to children constantly scrutinizing new concepts to understand the world, helps us ask ourselves: Is this the way we should take at all? Why do we need it, or should we let it off anyhow.

This article treats the Umwelt as a communication system grounded in psychology. It is a system that can be taken apart through analysis and tested through observation.

As stated above, we build our ideas on von Uexküll’s view that living beings deal not with an objective setting but with a subjective, meaning-filled realm. Consequently, the Communicative Umwelt is described here as a system composed of entities, signals, channels, and feedback loops. Together, these elements influence perception, interpretation, and communication, but also creativity, and, finally, and often missing, action.

Entities comprise the primary actors and structures involved in design processes, including (a) human agents such as designers, users, and other stakeholders; (b) material and symbolic artifacts such as products, tools, and interfaces; and (c) institutional structures such as markets, regulations, and cultural norms. This multi-entity perspective aligns with systemic accounts of design and innovation that emphasize interactions across human and non-human actors (Latour, 2005), while retaining a psychological focus on how these entities are subjectively perceived and interpreted (Döbler and Carbon, 2023).

Signals refer to the perceptual and symbolic information that entities emit; signals are quite compatible with ideas around affordances (Gibson, 1977; Koffka, 1935), especially with ideas of affordances from the domain of design (Carbon, 2019; Norman, 1988). They cover sensory perceptual cues (e.g., form, materiality, usability, affordance-related hints), symbolic cues (e.g., values, stories, sustainability claims, aesthetic codes), and social cues (e.g., norms, reputational markers, observed behavior of others—social interchange as stated in the model of Figure 1). Signals lack meaning by themselves. Only interpretation inside a specific Umwelt lets them matter (Krippendorff, 2005).

Channels map the paths along which signals move and are handled. They include sensory channels (mostly visual, haptic, auditory), social communicative channels (language, media, interpersonal exchange), and economic institutional channels (pricing, availability, incentives, legal frameworks). A signal succeeds or fails depending on how well it fits the channels that belong to a given Umwelt.

Feedback loops show how actions based on read signals loop back and change the Umwelt in return. Short-term feedback appears in direct user experience, acceptance, rejection, or tweaking, updating, reframing of products. Long-term feedback builds through habit formation, cultural settling, market reshaping, Zeitgeist effects (Carbon, 2010) and institutional change. In each time frame, feedback links personal perception and conduct to collective and systemic outcomes (Geels, 2002).

This operational move lets the Communicative Umwelt act as a mid-level theoretical bridge that connects psychological processes like perception, meaning-making, creativity, and acceptance with outcomes that matter for design. Each component remains open to study through well-known psychological and design methods that include perceptual experiments, longitudinal acceptance tracking (e.g., Repeated Evaluation Techniques), field studies, and comparative analyses of local design ecosystems. In this manner, the Umwelt becomes both an aid for interpretation and a guide for specific design decisions, including checks on their links to sustainability.

This Communicative Umwelt is highly dynamic, arousing, triggering and inspiring. And it is also tolerant. This tolerance to new ideas is essential. Ideally, we should think of a two-stage process: First we have to create the optimal conditions to generate as many as possible creative ideas, including radical innovations. I call it “essential” to tolerate these ideas, because we need flow experiences of the involved persons. Prior experience, knowledge, and information must be left out at this point, as they could inhibit potentially useful ideas from the beginning. It is in the second stage that they come into play and where an evaluation of the ideas takes place, for instance, based on the eco-balance to know whether the generated idea is beneficial and fits into a larger system.

## 4 Short excursus on measuring the acceptance of generated design solutions

The present paper clearly focuses on the first stage, so I will only briefly refer to some possibilities to address the second stage and will only refer to the acceptance of consumers to find solutions and how to measure this. Different methods exist to find out what humans need and want to use. An often-used but quite problematic technique is to ask people directly, so to say in an explicit way. The problem arises because most people are neither be able to tell about their fundamental needs nor about their specific needs for products. We can also ask them indirectly via so-called implicit measures such as the widely used implicit association test (IAT, see Greenwald et al., 1998), which is, however, only capable of measuring one dimension, typically the valence (positive vs. negative); other, multidimensional applications of the IAT, e.g., the so-called multi-dimensional IAT (md-IAT, see Gattol et al., 2011), have been developed meanwhile, and they show high reliability. All these procedures are quite good if you already have concrete exemplars to show, for instance, designed products or at least design concepts that can be presented to the respondent. These procedures are not of specific help if you aim to take the important step back where we want to learn about the deeper needs and attitudes of typical customers: Do they really need the brand new function, do they really use the product in the future, and even more fundamentally, do they really need this total new product at all? We can employ a mixture of techniques to get an idea of these important questions. First of all, humans typically base their assessments and do orient their evaluations towards design concepts, products and the typical usage they know from everyday life. This means they orient it to their present Umwelt which is mostly not a very innovative one, and which is linearly progressing with the newly experienced items and their usages in the Umwelt. Substantially changes in an Umwelt, however, do hardly ever happen. So, the according acceptance of substantially changed approaches to new products will be low at first stance. One way to reduce this problem is to employ a Repeated Evaluation Technique (RET, Carbon and Leder, 2005) before people have to assess the qualities, and especially the acceptance, of new design products. The RET forces people to evaluate given material on a pre-defined set of properties (Carbon, 2015) via a standard procedure of familiarization; this helps to get knowledge on the valence, acceptance, and other variables which are important in order to know how or whether at all the products will be used in the future.

## 5 Zeitgeist and Umwelt

The essential problem of creating substantially^2^ new ideas or products based on such ideas is that humans, and thus also creative minds, live in today’s world with a specific Zeitgeist signature. The Zeitgeist is a potential problem as the context, the mindsets and general approaches are very strongly dependent on the Zeitgeist. So the Zeitgeist creates a great portion of a creative mind’s specific “Umwelt” (von Uexküll, 1909). It is quite difficult to escape such an Umwelt and to switch to substantially new ideas that go beyond one’s Umwelt.

The concept of Umwelt, coined by Jakob von Uexküll in the early 20th century, implies that we are living, perceiving and thinking in a self-centred world representing the outer world. However, as the Zeitgeist-dependent qualities of the Umwelt are accompanied by further factors which are related to the individual, we can develop means to fundamentally change the Umwelt to be more suitably shaped to think differently, to create substantially different ideas. Such individual-related factors are, for instance, the specific learning history, individual experiences, and domains of expertise. It is clear that even these factors are inherently influenced, potentially even determined, by Zeitgeist, so they seem rather fixed. We can, however, specifically change them by using some techniques that will be referred to in the following, for the sake of escaping from the Zeitgeist.

## 6 The adequate Umwelt

What is an adequate Umwelt that optimally assists the designer in creating substantially new creative ideas? Let us think of the target domain of sustainable products which should optimally fit into a new consumer market order of ecologically driven, potentially emission-free and healthy products. We need a fundamentally different Umwelt from today’s typically available Umwelten (one environment allows for multiple “Umwelten” with multiple interpretations and perceptions related to our needs, so with one word, Umwelten are neither fixed nor evidently stable). In a nowadays world, most people are very consumption-driven, visceral needs have to be satisfied fast and in a full sense, and the products should be available globally, 24/7, and in every amount. As many research streams indicate, such a way of consumption attitude and behavior is not sustainable on the long run regarding many aspects, among others, ecological, ethical, health-related as well as economic reasons. General approaches demand more regionally produced material (Reidsma and Ewert, 2008), the use of easily available material including seasonal products (McMichael et al., 2007) when natural base material is deployed, and a fundamentally different understanding of how long a span of product life we should aim for (Favi et al., 2018) and how we repair or reuse (Appleby et al., 2010; but see Cooper and Gutowski, 2017) the originating material after product life has reached (Jacobs et al., 2018).

In typical Umwelten that does not fundamentally address the mentioned issues of sustainability, we will only create products that show components or parts heading in this direction—most probably, they will not fit into a holistic concept for the future. Consequently, such solutions may only be niche products with doubtful success and market penetration, unsatisfactorily fulfilling the requirements defined by climate experts. Market research persistently tells us that consumers do not want to change their common, habituated behavior (e.g., Wuepper et al., 2019), even if the attitude towards such a change is positive (Yamoah and Acquaye, 2019), for instance, to be able to access a great variety of products (different bicycles for different, very specific purposes), the brand new product (Collart and Interis, 2018), and a high number of exemplars of one type at the same time (e.g., many computers in one single household). Conscious restrictions of consumption behavior are quite rare and unpopular, and mostly ideologically shaped, which does not resonate with common consumption attitudes. Politicians aiming towards such orientations are mostly rejected (e.g., the idea of a meat tax in Germany to move away from animal-based food; or taxing the fuel prices in France as an effort to move away from fossil fuels). Cognitive psychology, very much in contrast to market research, has founded knowledge and interest in humans’ essential needs and requirements. Main reason for this is, beyond relying on strong theory and methods, that psychology as a fundamental science is typically not a part of the economic system which ultimately requests increased consumption to increase the overall profit, e.g., by lowering the R&D costs per sold item. This independent role gives the opportunity to systematically analyze and test what humans really need and request when they are not primarily seen, informed and treated as mass consumers being part of a global market.

## 7 Learning from the past

With the very same product, coffee, the traditional coffee house culture took a very different direction and we can still learn from this example. In fact, this cultural way of intaking a coffee was a successful way to increase the level of socialization and communication, because the time to drink a coffee (and potentially eating accompanying baked products) was used for sitting together, and the time was eagerly extended by the demand characteristics of the high aesthetic ambience, by comfortable and practical seats and chairs (e.g., the Thonet chairs) and, last but not least, by a stylish set of plates filled and mounted with delicate regionally refined products. It is not surprising that in such Umwelten, a huge amount of creative minds came together to develop their general ideas shaping generations to come right there in the “Kaffeehaus” (e.g., writers like Stefan Zweig, politicians like Theodor Herzl, artists like Egon Schiele and architects like Adolf Loos). Quite recently, UNESCO even listed the “Viennese Coffee House Culture” as “Intangible Cultural Heritage”. Still, Viennese coffee houses are successful on the market, and they work as areas of communication, socializing, and immediate enjoyment. They still lack a long list of products, typically do not offer global goods, lack high standards of technology and refrain from going towards a “to-go” mentality, as their asset is the slowing down of the everyday’s life pace—interestingly, we revealed in other contexts that such a slowing down of perception and cognitive acts has the power to support deep understanding and fundamental new views on challenging topics (Muth and Carbon, 2016; Muth et al., 2015). There are new initiatives from the end of the 20th century approaching similar goals, such as the slow-food movement founded by Carlo Petrini in Italy in 1986 where the sustainability of the goods and the celebration and appreciation of them are priority 1. Slow-food movement established a variety of fundamental principles to reach this goal by trusting in regional products, made and traded by small local businesses and which are based on an ethical buying and trade system (see for a German NGO addressing all these issues: *FreaksToTable*; https://www.freakstotable.com/).

## 8 New Umwelten, new markets

The creation of new Umwelten does not automatically result in new markets. Rather, markets emerge and stabilize only under specific psychological, cultural, and institutional conditions that align perception, meaning, and behavior across a sufficient number of actors. From the perspective of the Communicative Umwelt, markets can be understood as large-scale feedback systems in which shared interpretations, expectations, and practices become temporarily stabilized (Geels, 2002). Designing within a sustainability-oriented Umwelt—characterized by regional embeddedness, material transparency, and long-term usage cycles—can foster market formation when several mechanisms jointly operate.

First, such Umwelten enhance meaning alignment: products are not evaluated solely on price or novelty, but on perceived coherence with personal values, identity, and local context. Psychological research suggests that this alignment reduces cognitive dissonance and increases tolerance for higher upfront costs or reduced convenience when products are experienced as “right” rather than merely “cheap” or “efficient.”

Second, trust mechanisms play a central role. Locally embedded production, visible material origins, and identifiable actors shorten communicative distances between producers and consumers. This transparency can compensate for scale disadvantages by reducing uncertainty and perceived risk—factors known to strongly influence adoption decisions, particularly for unfamiliar or non-standard products.

Third, experiential quality functions as a reinforcing feedback mechanism. Products and services embedded in rich, socially meaningful Umwelten—such as historically established coffee house cultures or contemporary slow-food initiatives—do not merely fulfill functional needs but structure time, social interaction, and attention. These experiential dimensions can shift evaluation criteria away from quantity and immediacy towards durability, appreciation, and repeated engagement. Importantly, with this shift we do not face anymore the virulent problem that people feel a reduction of their needs or even a loss of usual goods and practices, but they gain quality.

However, the viability of such market formations is not universal. Boundary conditions include income stability, cultural norms regarding consumption, availability of alternative infrastructures, and supportive institutional frameworks (e.g., regulation, taxation, education)—see Gifford and Nilsson (2014). In contexts dominated by extreme price competition, weak consumer protection, or limited access to information, Umwelt-based design strategies may remain confined to niche markets or symbolic consumption.

Importantly, the present framework does not claim that “market power” simply resides in the hands of consumers in an unrestricted sense. Rather, it suggests that markets become responsive when shifts in consumer behavior are sufficiently coordinated and institutionally supported to alter feedback loops affecting production, distribution, and regulation. From this perspective, sustainable markets emerge not through isolated ethical choices, but through cumulative changes in shared Umwelten that reshape what is perceived as normal, desirable, and acceptable.

Accordingly, the Communicative Umwelt framework invites comparative and longitudinal research examining how different configurations of psychological meaning, design practice, and institutional support give rise to divergent market trajectories. New Umwelten can enable new markets—but only under identifiable and empirically testable conditions.

## 9 The new Umwelt for the designer of tomorrow

To sum up, we have learned that humans live in Umwelten, which frame their individual perceptions and interpretations of their environments. These Umwelten generate mindsets of the involved humans. Thus, not only the mindsets of consumers but also those of the creative people are generated by such Umwelten, which means that they also shape the thinking and development of the future. As the 21st century is substantially characterized by existential ecological problems on a global magnitude, we need accordingly substantial changes in our philosophy of entire markets, production and consumption of goods, and, so, also of design. Substantial changes mean fundamentally different approaches to the whole ecosystem, not only minor or partial adjustments. Insights based on solid information about the status and the need for a change are helpful, but primarily, such cognitive processes need a very long period of time to transform into action, at least when we want to reach a wide range of people. Therefore, we need new, attractive offers from designers who generate creative products that are so appealing that common alternatives become outdated, old-fashioned, or even distressing. In order to create such high-quality solutions, we need a new Umwelt for the designer of tomorrow. This new Umwelt restricts itself to regional material and constraints the thinking in terms of complete usage cycles where products fit together and where products are not designed to compensate for the inadequacy of another product. Such an Umwelt is connected with the ecosystem and the labor force of humans and tries to find the most adequate solutions for the regional population, specifically. This new Umwelt thinks in local niches for best fits and is not about scaling things up to global products of dubious value and low matching performance with individuals’ real needs and requirements. Such an Umwelt will shape the mindset of the future!

## Data Availability

The original contributions presented in the study are included in the article/supplementary material, further inquiries can be directed to the corresponding author/s.

## Footnotes

1 On the website https://www.footprintnetwork.org, you can calculate your own ecological footprint revealing how many Earths would be needed if everyone lived like you including the personal Earth Overshoot Day indicating at which date of year we would have used as much natural resources the Earth can renew over the whole year, if everybody would live like you.

2 Whether such substantially new ideas should be called “disruptive” is a matter of discussion. I go here for the more general concept that the solutions found should be substantially different from existing ones. They should show a new direction or quality. They follow different approaches and are based on different frameworks than existing ones. It can even be that they refer to ideas which had already existed before and were answers to different questions in different times, but are now adequate to address nowadays challenges. Therefore, I explicitly prevent the usage of the term “disruptive.”
